# Comparison of CBERS-04, GF-1, and GF-2 Satellite Panchromatic Images for Mapping Quasi-Circular Vegetation Patches in the Yellow River Delta, China

**DOI:** 10.3390/s18082733

**Published:** 2018-08-20

**Authors:** Qingsheng Liu, Chong Huang, Gaohuan Liu, Bowei Yu

**Affiliations:** 1 State Key Laboratory of Resources and Environmental Information System, Institute of Geographic Sciences and Natural Resources Research, Chinese Academy of Sciences, Beijing 100101, China; huangch@lreis.ac.cn (C.H.); liugh@lreis.ac.cn (G.L.); yubw@lreis.ac.cn (B.Y.); 2 Jiangsu Center for Collaborative Innovation in Geographical Information Resource Development and Application, Nanjing 210023, China; 3 College of Resources and Environment, University of Chinese Academy of Sciences, Beijing 100049, China

**Keywords:** vegetation patch, CBERS-04, GF-1, GF-2, K-Means, example-based feature extraction

## Abstract

Vegetation in arid and semi-arid regions frequently exists in patches, which can be effectively mapped by remote sensing. However, not all satellite images are suitable to detect the decametric-scale vegetation patches because of low spatial resolution. This study compared the capability of the first Gaofen Satellite (GF-1), the second Gaofen Satellite (GF-2), and China-Brazil Earth Resource Satellite 4 (CBERS-04) panchromatic images for mapping quasi-circular vegetation patches (QVPs) with K-Means (KM) and object-based example-based feature extraction with support vector machine classification (OEFE) in the Yellow River Delta, China. Both approaches provide relatively high classification accuracy with GF-2. For all five images, the root mean square errors (RMSEs) for area, perimeter, and perimeter/area ratio were smaller using the KM than the OEFE, indicating that the results from the KM are more similar to ground truth. Although the mapped results of the QVPs from finer-spatial resolution images appeared more accurate, accuracy improvement in terms of QVP area, perimeter, and perimeter/area ratio was limited, and most of the QVPs detected only by finer-spatial resolution imagery had a more than 40% difference with the actual QVPs in these three parameters. Compared with the KM approach, the OEFE approach performed better for vegetation patch shape description. Coupling the CBERS-04 with the OEFE approach could suitably map the QVPs (overall accuracy 75.3%). This is important for ecological protection managers concerned about cost-effectiveness between image spatial resolution and mapping the QVPs.

## 1. Introduction

In arid and semi-arid zones, which comprise over 30% of the world’s land surface, vegetation often forms a pattern of patches with high plant cover interspersed with low plant cover or bare soil [1,2,3]. Because these vegetation patches are often difficult to discover at ground level, they were initially identified by the use of aerial photographs in the early 1940s [4]. Banded vegetation has been reported in Central America, Africa, and Australia, where annual rainfall is low (50–750 mm) [5,6,7,8,9], and spotted vegetation has been described in Africa, America, and Asia [10,11,12,13], which does not appear to be specific to particular soils, parent materials, or plant species [3]. There is an ongoing debate on the formation mechanisms of vegetation patch patterns [14,15,16,17,18,19]. In general, it is acknowledged that water reallocation is a key to the establishment of patches. Vegetation patchiness can reallocate the distribution of limited water, nutrients, and seeds, enhance primary production, affect species diversity, and constitute a key factor in ecosystem function, imminent catastrophic ecological shifts, such as desertification, and the management of arid and semi-arid lands [1,20,21].

Timely, reliable information on vegetation patches is necessary to quantify the effects of complex, interacting ecological processes and to formulate an understanding of ecosystem dynamics. Although traditional ground-based methods have revealed vegetation patch landscapes, the disadvantages of such methods include the limited spatial extent covered and the associated time and cost required [22,23]. Moreover, even intensive and long-term field observations cannot by themselves provide full coverage of dynamic variations in vegetation spatial patterns and the timing of changes in related driving forces [24]. Remote sensing can be used to map vegetation patch patterns with extended spatial and temporal coverage and thus has the potential to greatly improve our understanding of vegetation pattern dynamics in arid and semi-arid landscapes [21,24]. Aerial photographs are mostly used to study vegetation patch dynamics because of the higher spatial resolution, little affected by weather and longer historical archiving than satellite images [25]. Frenkel and Boss [26] monitored the establishment and exponential spread of distinctive circular *Spartina patens* patches on Cox Island, Canada, using sequential aerial photographs from 1939 to 1981. Kadmon and Harari-Kremer [27] used historical aerial photographs to study long-term vegetation dynamics. Becker and Getzin [28] described the distribution of fairy circles in Kaokoland, Namibia, based on a set of aerial photographs (scale 1:78000). Strand et al. [29] used 1 m panchromatic aerial photography to automatically detect plant spatial patterns. Barbier et al. [18] concluded that it was sufficient to study decametric-scale vegetation patch patterns using diachronic aerial photographs with a pixel size of 2 m. 

The advantages of aerial photography include high spatial resolution and the possibility to obtain the historical archiving data covered the certain regions acquired a long time ago. However, when mapping vegetation patch origins, it can be difficult to acquire historical aerial photographs for a given region because of limited historical coverage. With the rapid development of unmanned aerial vehicle (UAV) technology in the recent years, UAV has become a promising means for remote sensing of vegetation information [30]. However, to acquire aerial photographs over the large areas is still often expensive and not easy to operate for most users, especially compared with satellite remote sensing data. Satellite remote sensing data, such as Landsat Thematic Mapper (TM), Landsat Thematic Mapper Plus (ETM+), Systeme Probatoire d’Observation de la Terre (SPOT) images, Advanced Land Observing Satellite (ALOS), QuickBird (QB), Pleiades and ALOS PALSAR, have been widely used in vegetation community studies in arid and semi-arid environments. Laliberte et al. [31] used an object-oriented image analysis (OB) to map shrub encroachment from 1937 to 2003 in southern New Mexico using aerial photographs acquired between 1937 and 1996 and the QB satellite images acquired in 2003. Multi-temporal Landsat TM, SPOT 5, ALOS, ZY-3, and QB images have been used to monitor quasi-circular vegetation patch (QVP) recovery in abandoned land in the Yellow River Delta (YRD) [13,23,32,33,34,35]. It was a practical method to detect the location and dynamics of the QVPs through the analysis of the absorption position and depth using the tasseled cap transformation brightness from 15 m fusion-ready Landsat 7 ETM+ at-satellite reflectance [34]. The QVPs could be effectively detected using the edge detector and the geometric differences between the QVPs and background based on SPOT 5 (2.5 m resolution fusion imagery), ALOS (2.5 m resolution fusion imagery) and ZY-3 (5.8 m resolution multispectral imagery) images, and the detection accuracy was 93.4%, 89.3% and 64.1%, respectively [23,32,33]. The object-based approach was a useful tool for mapping the QVPs with the QB high spatial resolution multispectral image, and the precision rate was 65.6% [35]. Pu et al. [36] evaluated the potential of multi-seasonal high resolution Pleiades satellite imagery (0.5 m resolution fusion imagery) for mapping urban tree species using random forest (RF), support vector machine (SVM) and linear discriminant analysis (LDA) classifiers, and found that the seasonal effect on tree species classification was statistically significant and the RF outperformed both the SVM and LDA although they had resulted in relatively low average accuracy of tree species classification. Pham et al. [37] mapped the spatial distribution of mangrove species using the ALOS PALSAR data and an optimized rule-based logistic model tree algorithm, and found the overall accuracy of 2015 was higher than that of 2010 which may be due to the fact that ALOS-2 PALSAR imagery acquired for 2015 (6.25 m pixel spacing) has better spatial resolution than ALOS PALSAR imagery for 2010 (12.5 m pixel spacing).

The available satellite remote sensing platforms have different spectral (1 nm to 100 nm), spatial (0.5 m to 1 km), and temporal (0.5 day to 1 month) resolutions. However, not all remote sensing images are suitable for mapping decametric-scale vegetation patches and studying vegetation patch pattern dynamics. Spatial scale and spectral resolution play important roles, and the necessity of compromise between spatial and spectral resolution and classification accuracy is widely accepted, as described below [22]. In general, image spatial resolution significantly impacts the ability to accurately map vegetation properties [38]. As spatial resolution increases, the accuracy with which small vegetation patches can be mapped and characterized increases. However, the cost of acquiring fine-resolution remote sensing data and the associated data processing time over large land areas is prohibitive. Therefore, ongoing research is devoted to the comparison of the mapping and detection capabilities of various sensors to determine the optimal scale for monitoring vegetation, burn severity, land surface water, gross primary productivity, soil information, sand dune features, and archaeological crop marks, amongst others [22,38,39,40,41,42,43,44,45,46,47,48,49,50,51,52,53,54]. The overall success rate for mapping the six invasive plants using full resolution Advanced Visible Infrared Imaging Spectrometer (AVIRIS, 174 bands, 4 m spatial resolution) was 17% more than that of the spatially degraded AVIRIS (174 bands, 30 m) [22]. The AVIRIS images at spatial resolution 20 through 60 m were acceptable for identifying ecosystem-level dominant species using linear discriminant analysis, but the best accuracies were obtained at 40 m resolution, which should be compared using other pixel- and object-based classifiers to gain a broader understanding of the possible affect of spatial resolution on species mapping [38]. Compared with the SPOT 5 imagery, the QB imagery had a higher thematic mapping accuracy for tree cover in savannas, independent of mapping techniques (normalized difference vegetation index threshold approach and object-based image analysis method), but vegetation patch size distributions mapped with object-based image analysis of SPOT 5 were similar to that of the QB mapping, which indicated that SPOT 5 image was suitable for mapping tree cover patterns at regional scale [46]. The most cost-effective option for *Lantana camara* L. mapping was provided by Landsat TM (30 m) with no significant difference in overall accuracies between the Landsat TM, SPOT 5 (10 m) and QB (2.4 m) imagery [48]. According to the desired requirements, the WorldView-2 image (2 m) was good choice to obtain a global estimation of giant reed invasion, but when local maps of giant reed invasions were necessary, airborne multispectral imagery (0.5 m) was desirable [51]. Consequently, it is important to compare the different types of satellite images that are available for vegetation patch studies, especially images from high-resolution (fine–spatial resolution) satellite sensors. Although the previous research had demonstrated that the images with a pixel size of 2 m were suitable for mapping vegetation patch patterns [18,23,33], if the same vegetation patch can be detected at a coarser spatial resolution, future studies on vegetation patch patterns may avoid the cost-, time-, and labor-intensiveness associated with high–spatial resolution remote sensing images, unless detailed analytical data are needed.

The recent launch of several Chinese high–spatial resolution satellite sensors, which feature pixel sizes of 0.8–10 m, provides a new opportunity to compare the accuracy of satellite images with different resolutions for mapping vegetation patches. Although the majority of satellite images from these sensors are free to registered users, the images from some satellites, such as the second Gaofen Satellite (GF-2), is free only to authorized users, and payment for acquisition must be made by other users. Moreover, due to the limited revisit period and swath width of high–spatial resolution satellite sensors and other effects, such as cloud coverage, it is often beneficial to apply various viable spatial resolution images from the different sensors. Therefore, it is also necessary to evaluate the detection capabilities of different sensors to reduce costs and meet the desired requirements of the situation. Liu et al. [55] compared the capabilities of the different spatial resolution bands of SPOT 5 for mapping QVPs, and found that overall success rate for detecting QVPs using SPOT 5 panchromatic imagery (2.5 m) was 15% higher than that of SPOT 5 multispectral imagery (4 bands, 10 m); however, by contrast, the overall accuracy of area estimation for QVPs from SPOT 5 panchromatic imagery was less than that of SPOT 5 multispectral imagery. Liu et al. [56] found that compared with ALOS fusion-ready imagery (2.5 m), SPOT 5 fusion-ready imagery (2.5 m) had a higher mapping accuracy for QVPs at Gudong Oil Field. Li et al. [57] compared the capabilities of the different spatial resolution (2.1 m, 3.5 m, 5.8 m) bands of ZY-3 for mapping QVPs, and found that the higher spatial resolution image had potential to provide higher detect accuracy for QVPs. Zhang et al. [58] compared the capabilities of SPOT 5 fusion-ready (2.5 m), ALOS sharpened (2.5 m), and ZY-3 multispectral image (5.8 m) in mapping QVPs in the YRD from band statistics, normalized difference vegetation index, information entropy and sharpness of image, and found that SPOT 5 had the highest classification accuracy, and the classification accuracy of ZY-3 was higher than that of ALOS. Liu et al. [59] conducted a preliminary exploration and compared the capability of detecting QVPs using two panchromatic images acquired in the spring of 2015 from the first Gaofen Satellite (GF-1; panchromatic image with 2 m ground spatial distance) and China–Brazil Earth Resource Satellite 4 (CBERS-04; panchromatic image with 5 m ground spatial distance) in the YRD. However, the abovementioned comparisons did not include sub-meter images, which are acquired by the majority of high-resolution satellites, such as IKONOS, QB, GeoEye, Worldview, and GF-2, and more work is needed to compare the usefulness of the different spatial resolution images for mapping the QVPs. Moreover, not only the number of vegetation patches, but also the area, perimeter and perimeter/area of vegetation patch derived from satellite imagery need to be evaluated and compared, which are key factors for landscape ecology evaluation, patch pattern dynamic analysis, ecological processes, and ecological function monitoring and modeling [60].

Following Liu et al. [55,56,59], Li et al. [57], and Zhang et al. [58], the aim of this study is to compare images from GF-1, GF-2, and CBERS-04 to determine whether similar QVPs in the YRD can be detected at different spatial resolutions and the most cost-effective sensors for mapping QVPs, which is significant because an improved understanding of the tradeoffs between the spatial resolution and mapping accuracy is necessary to link the cost and time and mapping requirements to the vegetation patch distribution, pattern and structure of the ecosystem of the YRD, China, which has degraded and needs to be restored. In addition, compared with the study by Liu et al. [59], the images acquired in the summer of 2016, experimental areas with different types of QVP and sub-meter GF-2 panchromatic image are analyzed in this study. An exhaustive comparison of the spatial and spectral characteristics of GF-1, GF-2, and CBERS-04 is beyond the scope of this paper. Instead, we focus on comparing the three types of images for mapping vegetation patches based on number, area, perimeter and perimeter/area of the QVPs from the following two perspectives, which should be useful for selecting the most cost-effective imagery to improve the ability of remote sensing monitoring vegetation status to aid ecosystem restoration and management in the YRD: (1) their classification accuracy using panchromatic images and the K-means unsupervised classification (KM) approach and (2) their classification accuracy using panchromatic images and the object-based example-based feature extraction with support vector machine classification (OEFE) approach.

## 2. Materials and Methods 

### 2.1. Study Area

**Figure 1:**
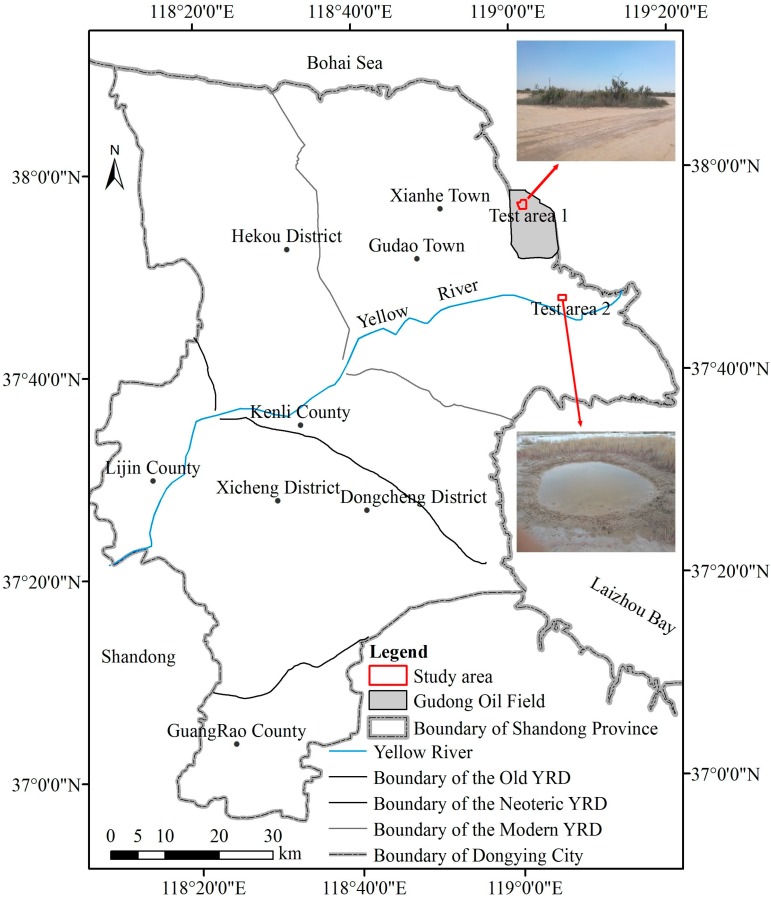
Location of the study area in the Yellow River Delta (YRD).

The study area comprises a section of the Gudong Oil field located within the YRD (118°7′ E–119°10′ E and 37°20′ N–38°10′ N) in Dongying City, Shandong Province, China (Figure 1). This region has been degraded and urgently needs restoration and has been focused on by the National Aeronautics and Space Administration (NASA) Gateway to Astronaut Photography of Earth (https://earthobservatory.nasa.gov/IOTD/view.php?id=4767). The area has a warm temperate continental monsoon climate, with an average annual temperature that varies from 11.7 °C to 12.1 °C and an annual average evaporation of 1962 mm. Mean annual precipitation ranges from 530 mm to 630 mm, 70% of which is recorded in the summer [61]. The study area has an aridity index of up to 3.56, qualifying it as an arid zone [13]. The soil texture of sediments in the YRD varies from sandy loam to silty clay [61]. *Suaeda salsa*, *Tamarix chinensis*, *and Phragmites australis* are the three common and widely distributed types of native vegetation that occur across the YRD [61]. QVPs are mainly composed of these species, with water or bare ground at their centers and have been discovered within an area of 25 km from the Bohai Sea [62]. According to the components, shape, and general structure of the vegetation communities, there are three types of QVP: (1) *Suaeda salsa* and water or bare ground at the center; (2) *Tamarix chinensis*, *Phragmites australis*, *Imperata cylindrica*, and *Atriplex patens* or *Limonium bicolor* (Bge.) e. Ktze encircled by a ring of *Suaeda salsa* and (3) *Tamarix chinensis* encircled by a ring of *Suaeda salsa* [13,62]. In this paper, two test areas covered by the different types of QVP encompass the regions characterized by QVPs with bare soil ground (Figure 1), which are good areas for comparing the different satellite images’ QVP detection capabilities. Test area 1 (2.24 km^2^) is located in abandoned land, where the QVPs are composed of *Suaeda salsa*, *Tamarix chinensis*, *Phragmites australis*, *Imperata cylindrica*, and *Atriplex patens* (photographs in Figure 1). Test area 2 (1.32 km^2^) covers tidal flats, where the QVPs are composed of *Suaeda salsa* and water or bare ground at the center (photographs in Figure 1).

### 2.2. Remote Sensing Data and Pre-Processing

CBERS-04 has four sensors, featuring (1) a 5 m resolution panchromatic band, three 10 m resolution multispectral bands, and a swath width of 60 km (Table 1); (2) four 20 m resolution multispectral bands with a swath width of 120 km; (3) three 40 m resolution infrared multispectral bands, an 80 m resolution thermal infrared spectral band, and a swath width of 120 km; and (4) a 73 m spatial resolution wide field imager with a swath width of 866 km [63].

GF-1 is equipped with (1) two multispectral scanners with a 2 m resolution panchromatic band, four 8 m resolution and a two-camera stitching swath width of 60 km (Table 1) and (2) four 16 m resolution multispectral bands with a four-camera stitching swath width of 800 km [64].

GF-2 is the first civil optical remote sensing satellite with a resolution higher than 1 m in China; the sub-satellite point spatial resolution reaches 0.8 m, and the satellite is equipped with two multispectral scanners, one featuring 0.8 m panchromatic detection and 4 m resolution multispectral observations and a two-camera stitching swath width of 45 km (Table 1) [65].

**Figure 2:**
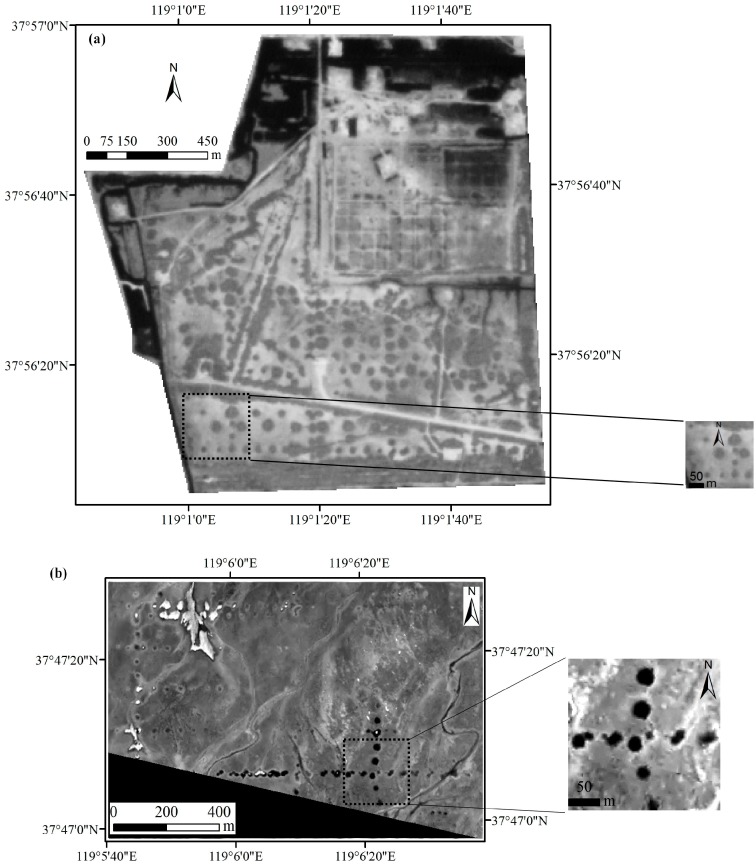
CBERS-04 (acquired on 10 July 2016) and GF-1 (acquired on 10 August 2016) panchromatic images covering test areas (**a**) 1 and (**b**) 2. The lower right images in (**a**) and (**b**) are the subset of the original size of CBERS-04 and GF-1 panchromatic images, respectively.

As far as possible, remote sensing images of vegetation growing season with similar acquisition dates were selected to be used to compare the capability of the different sensors for mapping the QVPs, which contributed to the identification of the QVPs and reduced the effects of atmospheric conditions, plant phenology, soil moisture and environmental changes due to the different image acquisition dates. After data query analysis on the archived images from CBERS-04, GF-1 and GF-2, this study used two CBERS-04 images acquired on 19 May and 10 July 2016, one GF-1 image acquired on 10 August 2016, and two GF-2 images acquired on 11 and 26 August 2016, respectively, which were downloaded from the China Centre for Resources Satellite Data and Application database (http://www.cresda.com). Because these images were not geometrically corrected, the CBERS-04 images were first geometrically registered to the corrected SPOT 5 image (2.5 m) using a quadratic polynomial nearest neighbor method with 24 GCPs collected by the image-to-image method (an average root mean square error of 0.4 pixels) in ENVI v5.1. Subsequently, the GF-1 and GF-2 images were geometrically registered to the corrected CBERS-04 image using a quadratic polynomial nearest neighbor method. To reduce calculation time, the image subsets covering test areas 1 and 2 were clipped to focus on the region of interest (Figure 2). Since the terrain of the study area is flat and the mapped results of the QVPs, not the images, were compared directly, the digital number values of the images were used directly without atmospheric correction and terrain correction. 

### 2.3. Vegetation Patch Classification

We focused on comparing the detectability of GF-2, GF-1, and CBERS-04 satellite images for mapping QVPs in the YRD to improve understanding of the tradeoffs between spatial resolution and mapping accuracy, which are critical to identifying cost- and time-efficient approaches for mapping vegetation patch distribution, pattern, and structure, but not for comparing the performance of different classification algorithms, although this is necessary and important [66,67]. However, to objectively assess the capabilities of the three types of images’ for mapping QVPs, the use of pixel- and object-based approaches was explored. Since only one panchromatic band was used for classification, the supervised classifiers of pixel-based approaches, such as the best-known maximum likelihood classification, vegetation index such as the NDVI, could not be used, and the known KM approach was selected to map QVPs in this study.

The KM approach iteratively clusters pixels into the nearest class using a minimum distance technique. Compared with supervised classifiers and object-based approaches, the KM approach is simple, which has widely been used to classify remote sensing images and evaluate the detection abilities of the different sensors [54,57,68]. Therefore, the KM approach (available in ENVI v5.1.) was used to classify vegetation patches from 0.8 m, 2 m, and 5 m resolution panchromatic images. The classification was performed using the following parameters: number of classes = 5, change threshold = 5%, and maximum iterations = 1. After classification, five classes were combined into two classes, the QVPs (including the QVPs and stripes) and Non QVPs (including water, bare soil, road and buildings).

Compared with pixel-based approaches, the OB technique is more locally adaptive; OB has increasingly been used to map vegetation patches and patterns and evaluate the detection abilities of different sensors [46,51,58,66,67]. Thus, the OEFE approach (available in ENVI v5.1.) was used to map vegetation patches in 0.8 m, 2 m, and 5 m resolution panchromatic images to evaluate whether QVPs can be identified using the edge algorithm embedded in OB. After repeated experiments, scale level (25) and merge level (75) were found to effectively delineate QVPs using the edge algorithm in CBERS-04 and GF-1 images; scale level (65) and merge level (95) were used for GF-2. To reduce processing time, an image mask was applied that included only the region of interest. A support vector machine (SVM) was used to perform example-based feature extraction, which has proven one of the most efficient machine learning techniques in estimating forest biomass and land-use and land-cover classification [69,70]. The QVPs from the QB imagery were randomly selected to create training and validation data for the SVM classification and accuracy assessment. The training and validation data for Non QVPs including water, bare soil, road and buildings were visually selected from GF-1 imagery referring to CBERS-04 and GF-2 imagery, which distributed in the whole imagery as evenly and typically as possible.

Accuracy assessments of the QVPs classification were performed based on the commonly used accuracy indices: overall accuracy (OA), Kappa coefficient, user’s accuracy (UA) and producer/s accuracy (PA). Only the vegetation class of the classification result imagery was exported in an ArcGIS shapefile vector format. After the stripes and small vegetation spots were removed through man-machine interactive operations based on the area and shape of the polygons, comparisons and assessments on the differences of area, perimeter and perimeter/area derived from CBERS-04, GF-1 and GF-2 imagery were performed. 

**Figure 3:**
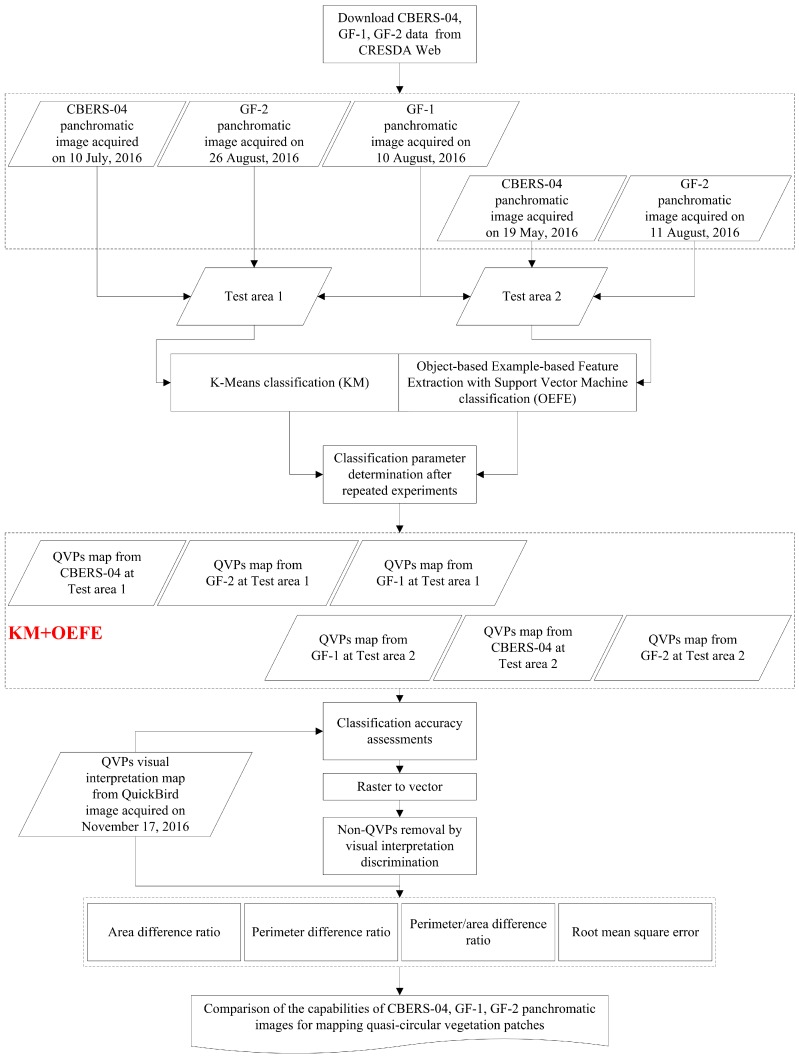
The flow chart of this research.

Then, the patch area difference ratio (Equation (1)), perimeter difference ratio (Equation (2)), and perimeter/area difference ratio (Equation (3)) were used to compare the QVPs in images extracted using each method with validation data from visual interpretation of the corrected QB image (which featured 0.6 m spatial resolution and was acquired on 17 November 2016); the root mean square error (RMSE) was used to determine whether the mapped QVPs were significantly different.

(1) $$Area\;difference\;ratio=\frac{A_{Ground\;Truth}-A_{i}}{A_{Ground\;Truth}}$$

(2) $$Perimeter\;difference\;ratio=\frac{P_{Ground\;Truth}-P_{i}}{P_{Ground\;Truth}}$$

(3) $$Perimeter/Area\;difference\;ratio=\frac{{PA}_{Ground\;Truth}-{PA}_{i}}{{PA}_{Ground\;Truth}}$$

where *A_GroundTruth_*, *P_GroundTruth_*, *PA_GroundTruth_*, and *Number_GroundTruth_* denote the area, perimeter, perimeter/area, and number of the QVP from the ground truth, respectively. *A_i_*, *P_i_*, and *PA_i_* represent the area, perimeter and perimeter/area of the correctly detected QVP from image *i*, where *i* indicates GF-1, GF-2, or CBERS-04. Figure 3 shows the whole flow chart of this research.

## 3. Results

Through visual interpretation of the QB image done in ENVI 5.1 software with a few field investigation data of the QVPs (investigated about 20 QVPs in test area 1 and 5 QVPs in test area 2 in July 2017) and multi-temporal finer spatial resolution images from Google Earth, we identified 137 and 109 QVPs distributed in test areas 1 and 2, respectively, which were used as validation or ground truth data to assess the accuracy of the classification. The perimeter/area of the QVPs was 0.08, 0.83, and 0.15 in test area 1, respectively, and 0.13, 0.76, and 0.27 in test area 2, respectively. The minimum, maximum, and mean area values of the QVPs were 19.49 m^2^, 3623.23 m^2^, and 872.87 m^2^ in test area 1, respectively, and 25.41 m^2^, 1426.12 m^2^, and 303.09 m^2^ in test area 2, respectively. Thus, the mean diameter of the QVPs in the study area was greater than 9.8 m, which showed that the QVPs could be detected using CBERS-04, GF-1 and GF-2 imagery with a pixel size of less than 5 m.

### 3.1. K-Means Unsupervised Classification

**Figure 4:**
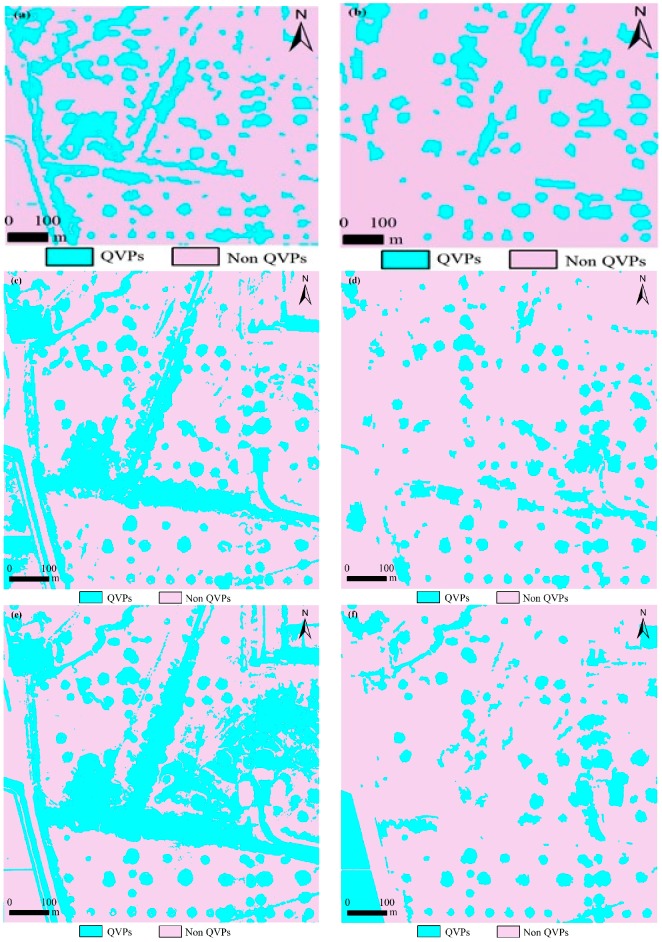
Mapping results of the QVPs, illustrated in the same small portion of the test area 1. (**a**) mapped from CBERS-04 imagery using K-Means (KM) classifier, (**b**) mapped from CBERS-04 imagery using object-based example-based feature extraction with support vector machine classification (OEFE) approach, (**c**) mapped from GF-1 imagery using KM classifier, (**d**) mapped from GF-1 imagery using OEFE approach, (**e**) mapped from GF-2 imagery using KM classifier, and (**f**) mapped from GF-2 imagery using OEFE approach.

**Figure 5:**
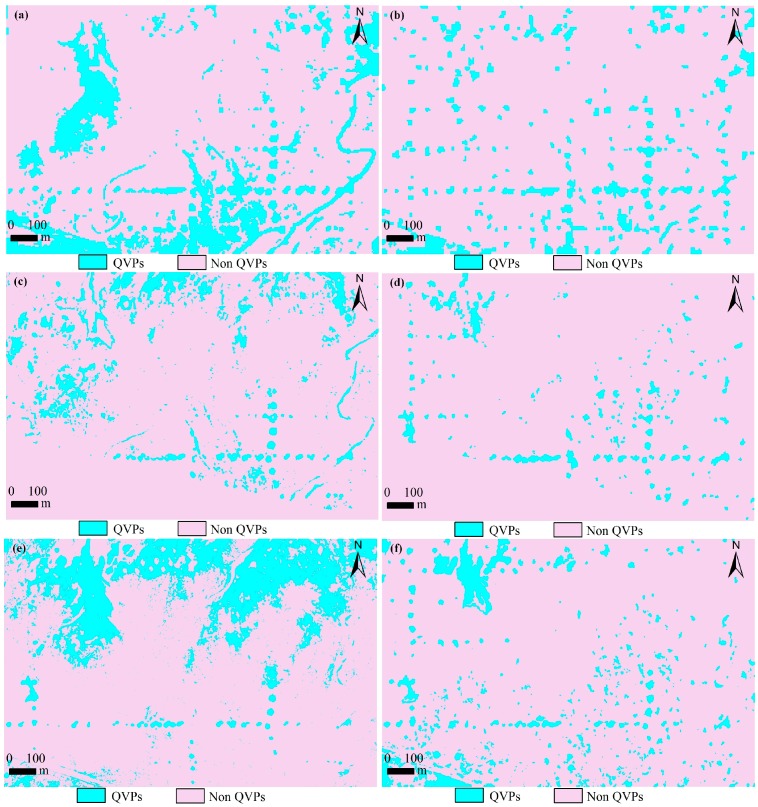
Mapping results of the QVPs in the test area 2. (**a**) mapped from CBERS-04 imagery using K-Means (KM) classifier, (**b**) mapped from CBERS-04 imagery using object-based example-based feature extraction with support vector machine classification (OEFE) approach, (**c**) mapped from GF-1 imagery using KM classifier, (**d**) mapped from GF-1 imagery using OEFE approach, (**e**) mapped from GF-2 imagery using KM classifier, and (**f**) mapped from GF-2 imagery using OEFE approach.

Table 2 summarizes the results of QVPs classification with the different images with the KM approach. The overall accuracy of CBERS-04, GF-1, and GF-2 classification images from K-Means classification was 74.7%, 70.5%, and 80.8%, and the kappa coefficient was 0.52, 0.42, and 0.62 in test area 1, respectively. In test area 2, the overall accuracy of CBERS-04, GF-1, and GF-2 classification images from K-Means classification was 77.9%, 73.6%, and 60.0%, and the kappa coefficient was 0.56, 0.46, and 0.19, respectively. Except for the classification result from GF-2 in test area 2, overall accuracies were acceptable. Figure 4a,c,e shows the QVPs mapping results created by the K-Means classifier for the same portion of the test area 1. Figure 5a,c,e shows the QVPs mapping results created by K-Means classifier for test area 2. Based on Table 2, Figure 4a,c,e and Figure 5a,c,e, the classification results of the QVPs created with CBERS-04 and GF-2 imagery appeared more accurate than those created with GF-1 imagery. 

Table 3 shows the RMSEs in area, perimeter and perimeter/area concerning the QVPs retrieved from GF-2, GF-1, and CBERS-04 images using the KM approach with those measured from the QB image. Of the detected QVPs from CBERS-04, GF-1, and GF-2 in test area 1, 67%, 97%, and 96%, respectively, had smaller area values than those measured from the visual interpretation of the QB image, whereas the same was true for 35%, 70% and 66% of the detected QVPs in test area 2, respectively. Of the detected QVPs from CBERS-04, GF-1, and GF-2 in test area 1, 33%, 29% and 9%, respectively, had smaller perimeter values than those measured from the visual interpretation of the QB image, whereas the same was true for 23%, 38%, and 18% of the detected QVPs in test area 2, respectively. Of the detected QVPs from CBERS-04, GF-1, and GF-2 in test area 1, 94%, 100%, and 100%, respectively, had greater perimeter/area values than those measured from the visual interpretation of the QB image, whereas the same was true for 63%, 93%, and 91% of the detected QVPs in test area 2, respectively. 

In order to compare the area and shape accuracy of the detected QVPs from the three satellite images more precisely, statistics based on 100 and 55 QVPs successfully detected from all three types of image in test areas 1 and 2, respectively, were analyzed: (1) In test areas 1 and 2, the cumulative percentage of the area difference ratio between −40% and 40% was greater for GF-2 than for GF-1 and CBERS-04. CBERS-04 and GF-2 had the largest cumulative percentage of area difference ratio between −20% and 20% in test area 1 (51%) and test area 2 (26%), respectively. (2) GF-1 had the largest cumulative percentage of perimeter difference ratio between −40% and 40% and between −20% and 20%, which was 94%, and 79% in test area 1, and 62% and 26% in test area 2, respectively. (3) Similar cumulative percentages of perimeter/area difference ratios were obtained from GF-1 and GF-2. The cumulative percentage of perimeter/area difference ratio between −40% and 40% and between −20% and 20% were the largest for CBERS-04 at the two test areas, which were approximately 30% higher than those of GF-1 and GF-2. (4) Based on the RMSE values (listed in Table 2), GF-2 resulted in the most accurate estimation on area, and GF-1 most accurately measured perimeter in test area 1, whereas CBERS-04 most accurately measured perimeter in test area 2. The RMSE values in perimeter/area from GF-2 and CBERS-04 were similar in test area 1, whereas CBERS-04 had a minimum RMSE value in test area 2. In test area 1, the RMSE values for area, perimeter, and perimeter/area were minimized between GF-1 and GF-2, and the area and perimeter/area obtained from CBERS-04 were more similar to those obtained from GF-2 than to those obtained from GF-1. For the similarity between CBERS-04, GF-1, and GF-2 in test area 2, the minimum RMSE values for area, perimeter, and perimeter/area were from between GF-1 and GF-2, between CBERS-04 and GF-1, and between CBERS-04 and GF-2, respectively.

### 3.2. Object-Based Example-Based Feature Extraction

Table 4 summarizes the results of QVPs classification with the different images with the OEFE approach. The overall accuracy of CBERS-04, GF-1, and GF-2 classification images from OEFE classification was 75.3%, 76.4%, and 75.6%, and the kappa coefficient was 0.53, 0.54, and 0.52 in test area 1, respectively. In test area 2, the overall accuracy of CBERS-04, GF-1, and GF-2 classification images from the OEFE approach was 67.0%, 75.7%, and 77.0%, and the kappa coefficient was 0.35, 0.51, and 0.53, respectively. Figure 4b,d,f shows the QVPs mapping results created by the OEFE approach for the same portion of the test area 1. Figure 5b,d,f shows the QVPs mapping results created by the OEFE approach for the test area 2. Based on Table 2, Figure 4b,d,f and Figure 5b,d,f, it is obvious that for the QVPs distributed in test area 1 the mapping results created by CBERS-04, GF-1 and GF-2 imagery with the OEFE approach were similar, and for the QVPs distributed in test area 2 the mapping results created by GF-1 and GF-2 imagery with the OEFE approach appear more accurate than that created with CBERS-04 imagery. 

Table 5 shows the RMSEs in area, perimeter and perimeter/area concerning the QVPs retrieved from GF-2, GF-1, and CBERS-04 images using example-based feature extraction with SVM classification with those measured from the QB image. Of the detected QVPs from CBERS-04, GF-1, and GF-2 in test area 1, 25%, 90%, and 96%, respectively, had smaller area values than those measured from the visual interpretation of the QB image, and the same was true for 61%, 84% and 61% of the detected QVPs in test area 2, respectively. Of the detected QVPs from CBERS-04, GF-1, and GF-2 in test area 1, 3%, 27%, and 9%, respectively, had smaller perimeter values than those measured from the visual interpretation of the QB image, and the same was true for 42%, 41%, and 13% of the detected QVPs in test area 2, respectively. Of the detected QVPs from CBERS-04, GF-1, and GF-2 in test area 1, 78%, 98%, and 100% respectively, had greater perimeter/area values than those measured from the visual interpretation of the QB image, and the same was true for 76%, 97%, and 14% of the detected QVPs in test area 2, respectively.

The 75 and 45 QVPs successfully detected from all three types of images from test areas 1 and 2, respectively, were analyzed: (1) In test area 1, the cumulative percentage of area difference ratio between −40% and 40% was greater for GF-2 than for GF-1 and CBERS-04, whereas that in test area 2 was greater for CBERS-04 than for GF-1 and GF-2. CBERS-04 and GF-2 had the largest cumulative percentage of area difference ratio between −20% and 20% in test area 2 (33%), whereas that of CBERS-04 was the largest in test area 1 (59%). (2) In the two test areas, GF-1 had the largest cumulative percentage of perimeter difference ratio between −40% and 40% and between −20% and 20% at 96% and 80% for test area 1 and 84% and 62% for test area 2, respectively. In test area 2, CBERS-04 had the same cumulative percentage of perimeter difference ratio between −20% and 20% as that of GF-1. (3) Similar cumulative percentages of perimeter/area difference ratios were obtained from GF-1 and GF-2. These ratios between −40% and 40% and between −20% and 20% were the largest for CBERS-04 at the two test areas, which was higher than those of GF-1 and GF-2. (4) Based on the RMSE values (listed in Table 3), GF-2 estimated area most accurately, and GF-1 measured perimeter most accurately. CBERS-04 had a minimum RMSE value in perimeter/area in the two areas. For the similarities between CBERS-04, GF-1, and GF-2 in test area 1, the RMSE values for area and perimeter were minimized between GF-1 and GF-2, and that in perimeter/area obtained from CBERS-04 was the smallest, whereas in test area 2 that in area was the smallest between GF-1 and GF-2. The RMSE value for perimeter was the smallest between CBERS-04 and GF-1, and the RMSE values for perimeter/area were similar for CBERS-04, GF-1, and GF-2.

## 4. Discussion

To date, few studies have been published comparing and evaluating results obtained from different satellite sensors by area, perimeter, and perimeter/area. Many researchers have compared the classification accuracy in the number of the detected targets. This study compared the results obtained from CBERS-04, GF-1, and GF-2 images by number, area, perimeter, and perimeter/area of QVPs. 

### 4.1. K-Means Unsupervised Classification and Object-Based Example-Based Feature Extraction Approaches

The KM approach has been widely used to classify remote sensing images, evaluate the detection capability of different sensors [54,71], and identify QVPs [57,59]. The OB approach is increasingly being used to map vegetation patches and patterns and evaluate the detection abilities of different sensors [46,51,58,59,66,67]. Boggs [46] found that the OB approach provided acceptable mapping accuracy using fine–spatial resolution QB images, and the OB approach was particularly important in tree canopy classification using relatively coarse-resolution SPOT 5 images. For the QVPs distributed in test area 1, where the QVPs are composed of plants and have a relatively high contrast with the surrounding environment, the mapped results of the QVPs from three different sensors’ images with the OEFE approach appear more consistent than those created with the KM classifier, which may be attributed to the training samples selection, that is to say, the training samples have important effects on the classification result created by the OEFE approach. For the QVPs distributed in test area 1, the different classifiers and the improvement of image spatial resolution do not have a clearly superior mapped result, which indicates that CBERS-04 imagery is sufficient for mapping this type of QVP distributed in this region, due to great contrast between the QVPs and background. For the QVPs distributed in test area 2, where the QVPs are composed of vegetation, bare soil and water, and affected by tidal water, and some QVPs have a relatively low contrast with the surrounding environment; the mapped results of the QVPs from GF-2 imagery with the OEFE approach appear more accurate than those created with the KM classifier, which indicates that the classification accuracy could be improved through more training samples. In general, except for the relatively low accuracy of GF-2 with the KM classifier (60.0%) in test area 2, the results indicated that the KM and OEFE approaches provided relatively high mapping accuracy of QVPs with CBERS-04, GF-1, and GF-2 images. Neither approach had a clearly superior detection result.

Table 3 and Table 5 showed that in test area 1, which had a higher QVP detection result, all three types of images with the KM approach produced lower RMSE values for area and perimeter compared to the ground truth than did the OEFE approach, which indicated that the QVP areas obtained using the former approach were more like the ground-truth areas obtained from visual interpretation. However, in the description of patch shape (perimeter/area), the OEFE approach outperformed the KM approach, especially in the relatively coarse-resolution GF-1 and CBERS-04 images. The KM approach produced lower RMSE values for area, perimeter, and perimeter/area between GF-1, GF-2, and CBERS-04 images than did the OEFE approach, which indicated that the former approach generated more comparable mapping results using the different images. On the other hand, the OEFE approach better delineated details in the various images. Some differences in the analysis results from the two test areas should be further evaluated in the future. 

In addition, the potential of the imagery itself may not be truly evaluated by the results from one or several classification methods [38], which are susceptible to expert experiences, the other pixel- and object-based methods should be used to evaluate the results obtained from different satellite sensors. In fact, the QVPs in the study area have the obvious geometric differences with the surrounding water, bare soil, road, buildings and stripes, and integrating edge detector with the algorithms for extracting circular and elliptical objects based on mathematical morphology may detect the QVPs better [23]. In recent years, random forest classifier and other machine learning algorithms also have proved to be highly successful in complex remote sensing applications with improved classification accuracies [36,72]. In the future, these approaches should be used to evaluate the mapped results of the QVPs from CBERS-04, GF-1 and GF-2 imagery.

### 4.2. Quasi-Circular Vegetation Patch Identification with GF-1, GF-2, and CBERS-04

The spatial resolution of the images should be smaller than the size of the target object [46]. The QVPs ranged from 2.9 m to 31.5 m in diameter, and the majority ranged from 10 m to 20 m in diameter [13], which is larger than the spatial resolutions of the GF-2, GF-1, and CBERS-04 panchromatic images (0.8 m, 2 m, and 5 m, respectively). Generally, image spatial resolution has a significant impact on the ability to accurately map vegetative properties [38]. As spatial resolution increases, the accuracy with which small objects are identified and characterized increases. However, the classification accuracy of internally homogeneous classes is not influenced by the spatial resolution of the image [48,73]. This is consistent with the results of this study that the QVP mapping accuracy was not increased with increased image spatial resolution from 5 m (CBERS-04) to 2 m (GF-1), especially for the QVPs in test area 1. 

**Figure 6:**
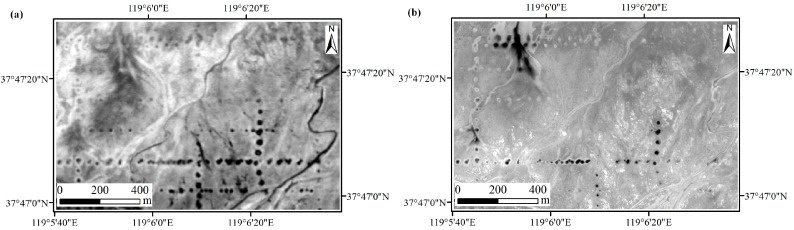
(**a**) CBERS-04 panchromatic image acquired on 19 May 2016 and (**b**) GF-2 panchromatic image acquired on 11 August 2016 for test area 2. Dark and bright quasi-circular objects are the QVPs.

The reasons for the different results in the two areas may be that the soil and QVPs in test area 2 were affected by tidal water from the Bohai Sea, which reduced the contrast between the QVPs and background, thus decreasing the detection result (Figure 2 and Figure 6); moreover, the bare spots in the upper right of the test area 2 had the similar spectral features with the neighbor bare soil which also decreased the detect result (Figure 6b), whereas test area 1 is seldom disturbed because of the cofferdam. For the QVPs in test area 2, if the images with the significant contrast between the QVPs and background could not be used, the higher spatial resolution image could not reach the higher classification accuracy for the QVPs.

The RMSE values for area (compared with the ground-truth area) were smallest for GF-2 using both classification approaches, whereas the RMSE values for perimeter/area were smallest for CBERS-04. The area and perimeter/area values of the QVPs obtained from CBERS-04 were more like those of GF-2 than those of GF-1. Therefore, CBERS-04 panchromatic images with a spatial resolution 5 m were sufficient for the detection of QVPs, rendering the purchase GF-1 panchromatic images unnecessary. In fact, many QVPs in the YRD are large enough to be discerned in 10 m SPOT images [55]. However, GF-2 may be better suited for the detection of details of QVPs, such as vegetation composition and structure.

The area of the minimum QVP detected by all three types of image was 111 m^2^. The areas of the QVPs undetected by CBERS-04 or GF-1 ranged from 156 m^2^ to 2033 m^2^, which indicated that the area of QVPs was not the factor influencing the detectability of CBERS-04 and GF-1. The detectability of images was affected by QVP conglutination, contrast between QVP and background within images, and QVP types, amongst others. For CBERS-04 images, 7 of 15 QVPs undetected by the OEFE approach could not be identified by the KM approach, whereas for GF-1 images, 14 of 23 QVPs undetected by the OEFE approach could not be identified by the KM approach, which indicated that the classification methods impacted the QVP detection rate. Four of seven QVPs had areas smaller than 225 m^2^ (less than 3 × 3 pixels), which indicated that CBERS-04 had limitations for mapping small QVPs regardless of classification methods. The results of this study indicate that the detection rate of the QVPs obtained from finer–spatial resolution images is higher than that of the relatively coarse–spatial resolution images, but accuracy improvement is limited from the area, perimeter, and perimeter/area of the QVPs.

Notably, the areas of the QVPs obtained via visual interpretation of QB images were greater than those obtained from all three types of image by both the KM and OEFE approaches. This phenomenon should be considered when assessing patch areas and dynamics using computer classification results and was likely attributable to the presence of *Suaeda salsa* at patch edges, *Tamarix chinensis* shadows within the patches, and patches with and without white salt-influenced regions. 

## 5. Conclusions

This study compared the capability of Chinese GF-1, GF-2, and CBERS-04 satellite images to detect the number, area, perimeter, ad perimeter/area of the QVPs in the YRD using the KM and the OEFE approaches. 

The results showed that GF-2 images with the KM approach performed best for QVP detection, and the OEFE approach performed best for vegetation patch shape description. Neither approach had a clearly superior QVP detection result. 

The QVP detection accuracy of finer–spatial resolution images was higher than that of relatively coarse–spatial resolution images. However, accuracy improvement in terms of QVP area, perimeter, and perimeter/area is limited. CBERS-04 panchromatic images with a spatial resolution of 5 m were sufficient for detecting QVPs at patch scale, rendering the purchase of GF-1 panchromatic images unnecessary when ecological protection managers consider the cost-effectiveness of image spatial resolution and actual requirements. GF-2 may be better used to detect QVP details, which are useful for mapping patch structures and components and studying patch formation mechanisms. Overall, coupling the CBERS-04 with the OEFE approach could suitably map the QVPs in the study area.

For the different QVPs in the different area, the optimal image acquisition date when the QVPs have the highest contrast with the surrounding environment is more important than the classification approaches and the improvement of image spatial resolution.

Although QVPs with an area of 111 m^2^ were detected by all three types of image, the QVPs with areas less than 225 m^2^ remained a challenge. To further investigate the potential of the Chinese GF-1, GF-2, and CBERS-04 satellite platforms for QVP mapping, more comprehensive sets of paired panchromatic images and fusion images (pan-sharpening panchromatic band with multispectral bands of these sensors) acquired in different growing seasons should be evaluated.

## Figures and Tables

**Table 1:** Image acquisition dates, spectral ranges, and spatial resolutions.

Satellite	Band No.	Spectral Range (nm)	Spatial Resolution (m)	Acquisition Date
CBERS-04	1	510–850	5	19 May and 10 July 2016
GF-1	1	450–900	2	10 August 2016
GF-2	1	450–900	0.8	11 and 26 August 2016

**Table 2:** Summary of classification results of the quasi-circular vegetation patches retrieved from GF-1, GF-2, and CBERS-04 images using K-Means classification.

Test Area	Class	CBERS-04		GF-1		GF-2	
		PA (%)	UA (%)	PA (%)	UA (%)	PA (%)	UA (%)
1	QVPs	58.57	97.16	62.71	81.76	77.62	87.61
	Non QVPs	97.56	62.35	81.11	61.69	85.14	73.76
	OA (%)	74.67		70.54		80.82	
	Kappa	0.52		0.42		0.62	
2	QVPs	59.3	95.83	46.89	97.46	39.34	64.34
	Non QVPs	97.3	69.57	98.84	66.23	79.49	58.22
	OA (%)	77.88		73.55		60.03	
	Kappa	0.56		0.46		0.19	

Note: PA = Producer’s accuracy, UA = User’s accuracy, OA = Overall accuracy, Kappa = Kappa coefficient, QVPs = Quasi-circular vegetation patches.

**Table 3:** The RMSEs in area, perimeter and perimeter/area concerning quasi-circular vegetation patches retrieved from GF-1, GF-2, and CBERS-04 images using K-Means classification.

Items	Test Areas	GF-2	GF-1	CBERS-04
The RMSE in area (m^2^)	1	243.68	330.18	352.18
	2	165.43	255.83	169.16
The RMSE in perimeter (m)	1	32.31	17.88	42.05
	2	52.16	45.37	32.88
The RMSE in perimeter/area	1	0.15	0.19	0.14
	2	0.36	0.57	0.13

**Table 4:** Summary of classification results of the quasi-circular vegetation patches retrieved from GF-1, GF-2, and CBERS-04 images using example-based feature extraction with SVM classification.

Test Area	Class	CBERS-04		GF-1		GF-2	
		PA (%)	UA (%)	PA (%)	UA (%)	PA (%)	UA (%)
1	QVPs	58.03	99.69	63.62	93.14	69.59	85.3
	Non QVPs	99.74	62.56	93.67	65.59	83.77	67.06
	OA (%)	75.25		76.4		75.62	
	Kappa	0.53		0.54		0.52	
2	QVPs	38.12	93.41	51.99	96.34	54.5	96.64
	Non QVPs	97.19	60.03	98.13	68.3	98.22	69.65
	OA (%)	66.99		75.67		77.03	
	Kappa	0.35		0.51		0.53	

Note: PA = Producer’s accuracy, UA = User’s accuracy, OA = Overall accuracy, Kappa = Kappa coefficient, QVPs = Quasi-circular vegetation patches.

**Table 5:** The RMSEs in area, perimeter and perimeter/area concerning quasi-circular vegetation patches retrieved from GF-1, GF-2, and CBERS-04 images using example-based feature extraction with SVM classification.

Items	Test Areas	GF-2	GF-1	CBERS-04
The RMSE in area (m^2^)	1	265.60	357.01	556.16
	2	183.13	182.52	224.87
The RMSE in perimeter (m)	1	37.55	27.90	50.38
	2	40.23	24.28	32.92
The RMSE in perimeter/area	1	0.21	0.10	0.04
	2	0.22	0.21	0.06

## References

[1] Aguiar M.R., Sala O.E. (1999). Patch structure, dynamics and implication for the functioning of arid ecosystem. Trends Ecol. Evol..

[2] Saco P.M., Willgoose G.R., Hancock G.R. (2007). Eco-geomorphology of banded vegetation patterns in arid and semi-arid regions. Hydrol. Earth Syst. Sci..

[3] Bordeu I., Clerc M.G., Couteron P., Lefever R., Tlidi M. (2016). Self-replication of localized vegetation patches in scarce environments. Sci. Rep..

[4] Lejeune O., Tlidi M., Lefever R. (2004). Vegetation spots and stripes: Dissipative structures in arid landscapes. Int. J. Quantum Chem..

[5] Valentin C., d’Herbes J.M., Poesen J. (1999). Soil and water components of banded vegetation patterns. Catena.

[6] Janeau J.L., Mauchamp A., Tarin G. (1999). The soil surface characteristics of vegetation stripes in Northern Mexico and their influences on the system hydrodynamics: An experimental approach. Catena.

[7] Galle S., Ehrmann M., Peugeot C. (1999). Water balance in a banded vegetation pattern: A case study of tiger bush in western Niger. Catena.

[8] Dunkerley D.L., Brown K.J. (1999). Banded vegetation near Broken Hill, Australia: Significance of surface roughness and soil physical properties. Catena.

[9] Dunkerley D.L., Brown K.J. (2002). Oblique vegetation banding in the Australian arid zone: Implications for theories of pattern evolution and maintenance. J. Arid Environ..

[10] Couteron P., Lejeune O. (2001). Periodic spotted patterns in semi-arid vegetation explained by a propagation-inhibition model. J. Ecol..

[11] Jankowitz W.J., Van Rooyen M.W., Shaw D., Kaumba J.S., Van Rooyen N. (2008). Mysterious circles in the Namib Desert. S. Afr. J. Bot..

[12] Armas C., Pugnaire F.I., Sala O.E. (2008). Patch structure dynamics and mechanisms of cyclical succession in a Patagonian steppe (Argentina). J. Arid Environ..

[13] Liu Q.S., Liu G.H., Huang C., Xie C.J., Bian F., Xie Y., Cui X., Zeng Y. (2013). Vegetation Patch Structure and Dynamics at Gudong Oil Field of the Yellow River Delta, China. Geo-Informatics in Resource Management and Sustainable Ecosystem.

[14] Sheffer E., Yizhaq H., Shachak M., Meron E. (2011). Mechanisms of vegetation-ring formation in water-limited systems. J. Theor. Biol..

[15] Kinast S., Ashkenazy Y., Meron E. (2014). A coupled vegetation–crust model for patchy landscapes. Pure Appl. Geophys..

[16] Sherratt J.A. (2005). An analysis of vegetation stripe formation in semi-arid landscapes. J. Math. Biol..

[17] Sherratt J.A. (2010). Pattern solutions of the Klausmeier Model for banded vegetation in semi-arid environments I. Nonlinearity.

[18] Barbier N., Couteron P., Lejoly J., Deblauwe V., Lejeune O. (2006). Self-organized vegetation patterning as a fingerprint of climate and human impact on semi-arid ecosystems. J. Ecol..

[19] D’Odorico P., Laio F., Ridolfi L. (2006). Patterns as indicators of productivity enhancement by facilitation and competition in dryland vegetation. J. Geophys. Res..

[20] Rietkerk M., Dekker S.C., De Ruiter P.C., Van de Koppel J. (2004). Self-organized patchiness and catastrophic shifts in ecosystems. Science.

[21] Von Hardenberg J., Kletter A.Y., Yizhaq H., Nathan J., Meron E. (2010). Periodic versus scale-free patterns in dryland vegetation. Proc. R. Soc. B.

[22] Underwood E.C., Ustin S.L., Ramirez C.M. (2007). A comparison of spatial and spectral image resolution for mapping invasive plants in coastal California. Environ. Manag..

[23] Liu Q.S., Liu G.H., Huang C., Xie C.J. (2014). Using SPOT 5 fusion-ready imagery to detect Chinese tamarisk (saltcedar) with mathematical morphological method. Int. J. Digit. Earth.

[24] Trodd N.M., Dougill A.J. (1998). Monitoring vegetation dynamics in semi-arid African rangelands: Use and limitations of Earth observation data to characterize vegetation structure. Appl. Geogr..

[25] Valta-Hulkkonen K., Kanninen A., Pellikka P. (2004). Remote sensing and GIS for detecting changes in the aquatic vegetation of a rehabilitated lake. Int. J. Remote Sens..

[26] Frenkel R.E., Boss T.R. (1998). Introduction, establishment and spread of *Spartina patens* on Cox Island, Siuslaw Estuary, Oregon. Wetlands.

[27] Kadmon R., Harari-Kremer R. (1999). Studying long-term vegetation dynamics using digital processing of historical aerial photographs. Remote Sens. Environ..

[28] Becker T., Getzin S. (2000). The fairy circles of Kaokoland (North-West Namibia) origin, distribution, and characteristics. Basic Appl. Ecol..

[29] Strand E.K., Smith A.M.S., Bunting S.C., Vierling L.A., Hann D.B., Gessler P.E. (2006). Wavelet estimation of plant spatial patterns in multitemporal aerial photography. Int. J. Remote Sens..

[30] Bryson M., Reid A., Ramos F., Sukkarieh S. (2010). Airborne vision-based mapping and classification of large farmland environments. J. Field Robot..

[31] Laliberte A.S., Rango A., Havstad K.M., Paris J.F., Beck R.F., McNeely R., Gonzalez A.L. (2004). Object-oriented image analysis for mapping shrub encroachment from 1937 to 2003 in southern New Mexico. Remote Sens. Environ..

[32] Liu Q.S., Liu G.H., Huang C., Xie C.J., Shi L. (2011). Using ALOS high spatial resolution image to detect vegetation patches. Procedia Environ. Sci..

[33] Liu Q.S., Zhang Y.J., Liu G.H., Huang C. (2013). Detection of Quasi-circular Vegetation Community Patches Using Circular Hough Transform Based on ZY-3 Satellite Image in the Yellow River Delta, China. Proceedings of the International Geoscience and Remote Sensing Symposium.

[34] Liu Q.S., Liu G.H., Huang C., Shi L., Zhao J. (2015). Monitoring vegetation recovery at abandoned land. Proceedings of the International Congress on Image and Signal Processing.

[35] Liu Q.S., Liang L., Liu G.H., Huang C., Li H., Zhao J. (2017). Mapping quasi-circular vegetation patches using QuickBird image with an object-based approach. Proceedings of the 10th International Congress on Image and Signal Processing, BioMedical Engineering and Informatics.

[36] Pu R.L., Landry S., Yu Q.Y. (2018). Assessing the potential of multi-seasonal high resolution Pleiades satellite imagery for mapping urban tree species. Int. J. Appl. Earth Obs. Geoinf..

[37] Pham T.D., Bui D.T., Yoshino K., Le N.N. (2018). Optimized rule-based logistic model tree algorithm for mapping mangrove species using ALOS PALSAR imagery and GIS in the tropical region. Environ. Earth Sci..

[38] Roth K.L., Roberts D.A., Dennison P.E., Peterson S.H., Alonzo M. (2015). The impact of spatial resolution on the classification of plant species and functional types within imaging spectrometer data. Remote Sens. Environ..

[39] Kunitomo J., Morimoto Y. (1999). Vegetation monitoring using different scale of remote sensing data. J. Environ. Sci..

[40] Wang L., Sousa W.P., Gong P., Biging G.S. (2004). Comparison of IKONOS and QuickBird images for mapping mangrove species on the Caribbean coast of Panama. Remote Sens. Environ..

[41] Wang J.B., Dong J.W., Liu J.Y., Huang M., Li G.C., Running S.W., Smith W.K., Harris W., Saigusa N., Kondo H. (2014). Comparison of gross primary productivity derived from GIMMS NDVI3g, GIMMS, and MODIS in Southeast Asia. Remote Sens..

[42] Griffith J.A., McKellip R.D., Morisette J.T. Comparison of Multiple Sensors for Identification and Mapping of Tamarisk in Western Colorado: Preliminary Findings. Proceedings of the ASPRS 2005 Annual Conference on Geospatial Goes Global: From Your Neighborhood to the Whole Planet.

[43] Alavi Panah S.K., Goossens R., Matinfar H.R., Mohamadi H., Ghadiri M., Irannegad H., Alikhah Asl M. (2008). The efficiency of landsat TM and ETM+ thermal data for extracting soil information in arid regions. J. Agric. Sci. Technol.-Iran.

[44] Selkowitz D.J. (2010). A comparison of multi-spectral, multi-angular, and multi-temporal remote sensing datasets for fractional shrub canopy mapping in Arctic Alaska. Remote Sens. Environ..

[45] Nagendra H., Rocchini D., Ghate R., Sharma B., Pareeth S. (2010). Assessing plant diversity in a dry tropical forest: Comparing the utility of Landsat and Ikonos satellite images. Remote Sens..

[46] Boggs G.S. (2010). Assessment of SPOT 5 and QuickBird remotely sensed imagery for mapping tree cover in savannas. Int. J. Appl. Earth Obs..

[47] Lozano F.J., Súarez-Seoane S., Luis E.D. (2010). Effects of wildfires on environmental variability: A comparative analysis using different spectral indices, patch metrics and thematic resolutions. Landsc. Ecol..

[48] Taylor S., Kumar L., Reid N. (2011). Accuracy comparison of Quickbird, Landsat TM and SPOT 5 imagery for *Lantana camara* mapping. J. Spat. Sci..

[49] Li W.B., Du Z.Q., Ling F., Zhou D.B., Wang H.L., Gui Y.N., Sun B.Y., Zhang X.M. (2013). A comparison of land surface water mapping using the normalized difference water index from TM, ETM+ and ALI. Remote Sens..

[50] Novack T., Esch T., Kux H., Stilla U. (2011). Machine learning comparison between WorldView-2 and QuickBird-2-simulated imagery regarding object-based urban land cover classification. Remote Sens..

[51] Fernandes M.R., Aguiar F.C., Silva J.M.N., Ferreira M.T., Pereira J.M.C. (2014). Optimal attributes for the object based detection of giant reed in riparian habitats: A comparative study between Airborne High Spatial Resolution and WorldView-2 imagery. Int. J. Appl. Earth Obs..

[52] Ghosh A., Fassnacht F.E., Joshi P.K., Koch B. (2014). A framework for mapping tree species combining hyperspectral and LiDAR data: Role of selected classifiers and sensor across three spatial scales. Int. J. Appl. Earth Obs..

[53] Agapiou A., Alexakis D.D., Hadjimitsis D.G. (2014). Spectral sensitivity of ALOS, ASTER, IKONOS, LANDSAT and SPOT satellite imagery intended for the detection of archaeological crop marks. Int. J. Digit. Earth.

[54] Els A., Merlo S., Knight J. Comparison of Two Satellite Imaging Platforms for Evaluating Sand Dune Migration in the Ubari Sand Sea (Libyan Fazzan). Proceedings of the 36th International Symposium on Remote Sensing of Environment.

[55] Liu Q.S., Liu G.H., Chu X.L. Comparison of Different Spatial Resolution Bands of SPOT 5 to Vegetation Community Patch Detection. Proceedings of the 2012 5th International Congress on Image and Signal Processing.

[56] Liu Q.S., Huang D., Liu G.H., Huang C. Remote Sensing and Mapping of Vegetation Community Patches at Gudong Oil Field, China: A comparative Use of SPOT 5 and ALOS data. Proceedings of the SPIE 8531.

[57] Li Y.Y., Liu Q.S., Liu G.H., Huang C. (2013). Detect Quasi-circular Vegetation Community Patches Using Images of Different Spatial Resolutions. Proceedings of the 6th International Congress on Image and Signal Processing.

[58] Zhang Y.J., Liu Q.S., Liu G.H., Tang S.J. (2015). Mapping of Circular or Elliptical Vegetation Community Patches: A Comparative Use of SPOT-5, ALOS and ZY-3 Imagery. Proceedings of the 8th International Congress on Image and Signal Processing.

[59] Liu Q.S., Liang L., Liu G.H., Huang C. Comparison of Two Satellite Imaging Platforms for Monitoring Quasi-circular Vegetation Patch in the Yellow River Delta, China. Proceedings of the SPIE 10405.

[60] Lafortezza R., Brown R.D. (2004). A framework for landscape ecological design of new patches in the rural landscape. Environ. Manag..

[61] Liu G.H., Drost H.J. (1997). Atlas of the Yellow River Delta.

[62] Liu Q.S., Liu G.H., Huang C., Wu C.S., Jing X. (2016). Remote sensing analysis on the spatial-temporal dynamics of quasi-circular vegetation patches in the Modern Yellow River Delta, China. Remote Sens. Technol. Appl..

[63] Cresda, CBERS-04, Slate. http://www.cresda.com/EN/satellite/7159.shtml.

[64] Cresda, GF-1, Slate. http://www.cresda.com/EN/satellite/7155.shtml.

[65] Cresda, GF-2, Slate. http://www.cresda.com/EN/satellite/7157.shtml.

[66] Kamal M., Phinn S. (2011). Hyperspectral data for mangrove species mapping: A comparison of pixel-based and object-based approach. Remote Sens..

[67] Ghosh A., Joshi P.K. (2014). A comparison of selected classification algorithms for mapping bamboo patches in lower Gangetic plains using very high resolution WorldView 2 imagery. Int. J. Appl. Earth Obs..

[68] Guo Y.S., Liu Q.S., Liu G.H., Huang C. (2016). Individual tree crown extraction of high resolution image based on marker-controlled watershed segmentation method. J. Geoinf. Sci..

[69] Liu Q.S., Liu G.H. Combining tasseled cap transformation with support vector machine to classify Landsat TM imagery data. Proceedings of the 6th International Conference on Natural Computation (ICNC 2010).

[70] Vafaei S., Soosani J., Adeli K., Fadaei H., Naghavi H., Pham T.D., Bui D.T. (2018). Improving accuracy estimation of forest aboveground biomass based on incorporation of ALOS-2 PALSAR-2 and Sentinel-2A imagery and machine learning: A case study of the Hyrcanian forest area (Iran). Remote Sens..

[71] Clark P.E., Seyfried M.S., Harris B. (2001). Intermountain plant community classification using Landsat TM and SPOT HRV data. J. Range Manag..

[72] Wang H., Zhao Y., Pu R.L., Zhang Z.Z. (2015). Mapping Robinia Pseudoacacia forest health conditions by using combined spectral, spatial and textural information extracted from IKONOS imagery and random forest classifier. Remote Sens..

[73] Cushnie J.L. (1987). The interactive effect of spatial resolution and degree of internal variability within land-cover types on classification accuracies. Int. J. Remote Sens..

